# Coagulation abnormalities following brown recluse spider (*Loxosceles reclusa*) envenomation: A description of 2 cases and review of the literature

**DOI:** 10.1093/ajcp/aqaf001

**Published:** 2025-01-30

**Authors:** Stephanie A Hart, David Gailani, Lorin A Bibb, Jeffrey P Zwerner, Garrett S Booth, Jeremy W Jacobs

**Affiliations:** Department of Pathology, Microbiology and Immunology, Vanderbilt University Medical Center, Nashville, Tennessee, US; Department of Pathology, Microbiology and Immunology, Vanderbilt University Medical Center, Nashville, Tennessee, US; Division of Pediatric Dermatology, Department of Dermatology, Vanderbilt University Medical Center, Nashville, Tennessee, US; Department of Dermatology, Vanderbilt University Medical Center, Nashville, Tennessee, US; Department of Pathology, Microbiology and Immunology, Vanderbilt University Medical Center, Nashville, Tennessee, US; Department of Pathology, Microbiology and Immunology, Vanderbilt University Medical Center, Nashville, Tennessee, US

**Keywords:** brown recluse spider, coagulopathy, coagulation, hemostasis, envenomation, loxoscelism, *Loxosceles reclusa*, toxicology

## Abstract

**Objective:**

Hemostatic abnormalities, including disseminated intravascular coagulation (DIC), are often cited as a common finding in patients following *Loxosceles* spider envenomation (ie, loxoscelism). The prevalence and severity of coagulopathy, however, particularly following *L reclusa* (ie, brown recluse) envenomation, is not well described. This study aimed to characterize coagulation laboratory parameters and coagulopathy in patients following *L reclusa* envenomation.

**Methods:**

We evaluated the coagulation laboratory parameters (eg, prothrombin time, partial thromboplastin time, coagulation factor activity levels, lupus anticoagulant [LA] testing) of 2 patients seen at our institution following brown recluse spider envenomation. We also comprehensively reviewed the literature for all reported cases of brown recluse spider envenomation and assessed patient demographics, clinical presentations, coagulation laboratory parameters, and outcomes.

**Results:**

We identified 2 patients with loxoscelism (1 cutaneous only, 1 systemic with hemolysis) with prolonged partial thromboplastin times but with normal clotting factor levels following envenomation. Literature review identified 263 patients: 12 patients had at least 1 prolonged clotting time, 31 reported a platelet count below 150 × 10^9^/L, and there was clinical concern for DIC in 12 cases. The odds of death were statistically significantly higher in patients with clinical concern for DIC than in cases without concern for DIC or coagulopathy (odds ratio, 82.9 [95% CI, 12.6-433.8]; *P* < .001).

**Conclusions:**

Following brown recluse spider envenomation, hemostatic perturbations are infrequent and clinical coagulopathy is uncommon, but the odds of death following a brown recluse spider bite are statistically significantly greater if DIC develops, even when compared to individuals with hemolysis without DIC.

**Key Points**

- The prevalence and severity of coagulation abnormalities following *L reclusa* (brown recluse spider) envenomation (loxoscelism) is unknown.
- Analysis of 263 cases in the literature demonstrated that coagulation laboratory abnormalities were infrequent; when present, however, they did not usually indicate an overt coagulopathy.
- Disseminated intravascular coagulation is rare, even in severe loxoscelism with hemolysis, but is associated with statistically significantly higher odds of mortality when present.

## INTRODUCTION

Approximately 40 000 species of spiders have been identified worldwide, though few cause clinically relevant disease in humans.^1,2^ In North America, spiders of the genera *Latrodectus* (eg, *L mactans*, or the black widow spider) and *Loxosceles* (eg, *L reclusa*, or the brown recluse spider) are 2 of the best known and clinically most important with regard to human envenomation.^3-5^ The prevailing species of *Loxosceles* spiders in North America include *L reclusa, L arizonica,* and *L deserta.* In South America, the most common species of *Loxosceles* are *L intermedia, L laeta,* and *L gaucho*. Rare cases of envenomation by *L rufescens* have been reported in Europe and South Africa.^6,7^

Although humans and spiders have co-existed for millennia, it has been less than a century since the discovery that the bite of *Loxosceles* spiders can result in both cutaneous and systemic sequelae (ie, loxoscelism), which in rare cases can be fatal. Although initially described in the literature by Caveness in 1872,^8^ the first proven cases of loxoscelism were reported in South America in the 1930s and 1940s^9,10^ and in North America in the 1950s.^11,12^ Cutaneous loxoscelism is characterized by erythema, edema, pain, and necrosis at or near the site of envenomation.^6^ Although the initial bite of *Loxosceles* spiders may be inconspicuous, up to 70% of envenomated individuals develop cutaneous manifestations.^6^ Conversely, systemic loxoscelism is considered rare, although there is marked variability in its reported frequency (ie, from <1% to approximately 25% of envenomated individuals), which may relate to the particular *Loxosceles* species as well as patient demographics.^6,7,13^ The signs and symptoms of systemic loxoscelism are heterogenous, ranging from fever, headache, and gastrointestinal distress to severe hemolysis with profound anemia and subsequent multiorgan failure.^6,7,14-17^

Hemolytic anemia has been characterized in numerous studies following *Loxosceles* envenomation, including for *L reclusa*, the most widespread *Loxosceles* species in the United States.^14-17^ Although frequently alluded to as a common finding in systemic loxoscelism,^18,19^ the prevalence and severity of alterations in hemostasis, including disseminated intravascular coagulation (DIC), is less well described. Some authors have suggested that DIC in loxoscelism is “more a myth than a reality.”^20^ The belief that DIC is characteristic of severe systemic loxoscelism arose following a 1972 case report^21^ and experimental data published in 1973,^22^ though case descriptions that include coagulation laboratory results are the exception in the scientific literature. Nevertheless, sphingomyelinase D in *Loxosceles* venom has been shown to modify the cell surface expression of various proteins, including thrombomodulin and endothelial protein C receptor, potentially affecting hemostasis and providing a possible mechanistic explanation for DIC development.^23,24^

One of the largest studies of coagulation parameters following *Loxosceles* envenomation involved 81 patients admitted to a Brazilian hospital in a region endemic for *L gaucho*.^25^ The authors found that fibrinogen was increased in 17 of 34 and 20 of 22 patients after 1 week and 2 weeks, respectively.^25^ In addition, thrombocytopenia occurred in 13 of 74 and 8 of 35 patients after 1 week and 2 weeks, respectively.^25^ Of 53 patients, 4 had an international normalized ratio (INR) greater than 1.3, while 7 of 53 had an INR less than 1.0.^25^ Of 51 patients, 11 had a partial thromboplastin time (PTT) ratio above 1.25 in week 1.^25^ Notably, none of the patients were diagnosed with DIC.^25^ In a second study, Lopes et al^20^ reviewed 120 cases of *Loxosceles* envenomation in the published literature and identified 2 cases of clinically described DIC, 5 cases of thrombocytopenia, and 9 cases with “coagulation changes.” This review did not, however, distinguish among *Loxosceles* species. Thus, a limitation of these 2 studies is that it is not known whether these findings can be generalized to envenomations in the United States, where the overwhelming majority are due to *L reclusa.*

With regard to *L reclusa*, Robinson et al^26^ retrospectively assessed 57 individuals with moderate to severe loxoscelism at a hospital in the endemic region for *L reclusa* (the midwestern and southern United States).^27^ The authors found that 18% of patients had coagulation defects and 9% had thrombocytopenia,^26^ but no further coagulation results were reported, nor was there a description of how coagulation defects were defined. Ultimately, despite the dogma that DIC and coagulation anomalies are characteristic of loxoscelism, there is a paucity of data for recipients of *Loxosceles* envenomation in the United States. Therefore, we aimed to further characterize these findings through a description of our experience with 2 recent cases of coagulation abnormalities in patients with loxoscelism in conjunction with a comprehensive review of coagulation parameters in all published cases of loxoscelism due to *L reclusa*.

## METHODS

### Case descriptions

We describe 2 patients with coagulation laboratory abnormalities following brown recluse spider envenomation. Coagulation testing was performed on the STA R Max (Diagnostica Stago). Testing included prothrombin time (PT [STA Neoplastine CI Plus reagent kit, Diagnostica Stago]), PTT (STA PTT-Automate reagent kit, Diagnostica Stago), thrombin time (STA-Thrombin 2 reagent kit, Diagnostica Stago), and fibrinogen (STA-Fibrinogen 5 reagent kit, Diagnostic Stago). Lupus anticoagulant testing was performed using the dilute Russell viper venom time (dRVVT [CRYOcheck LA Check and CRYOcheck LA Sure reagent kits, Precision BioLogic Incorporated]) and a hexagonal phase phospholipid neutralization assay (STA-Staclot LA, Diagnostica Stago). The results of the dRVVT screen are reported as the ratio of the patient’s result to the result of the pooled normal plasma (PNP). The results of the dRVVT assay confirmation are reported as a ratio of ratios by dividing the dRVVT screen ratio by the dRVVT confirmation ratio. The results of the STA-Staclot LA assay are reported as the delta of the screen and confirmation clotting times. The PT and PTT mixing studies were performed using CRYOcheck Pooled Normal Plasma (Precision BioLogic Incorporated) at 0 hours, after 1 hour of incubation at 37 °C, and after 2 hours of incubation at 37 °C. Coagulation factor activity levels were determined using STA factor-deficient plasmas (Diagnostica Stago). Complete blood cell counts were performed to assess hemoglobin values and platelet counts using the Sysmex XN-9000 system. Additional laboratory testing was performed according to manufacturer guidelines.

### Literature review

We performed a literature review to identify cases of brown recluse spider envenomation. We queried PubMed, Embase, and Web of Science from inception to October 3, 2024. The search strategies are shown in Table 1. We included all reported cases of brown recluse spider envenomation, irrespective of severity, provided that they (1) occurred within the spider’s known geographic range and (2) had at least 1 positive examination feature of brown recluse spider envenomation, as previously described.^2,28,29^ Cases of brown recluse spider envenomation are frequently reported outside their accepted endemic area but are exceedingly unlikely to be legitimate brown recluse spider bites.^30^ As such, if the bite occurred outside the spider’s endemic region, we included the case only if the spider was captured at the time of the bite and identified as *L reclusa* by an expert (ie, an entomologist, arachnologist, or toxicologist).

**Table 1:** Search Strategy for PubMed, Embase, and Web of Science

Service	Search string
PubMed	(“Spider Bites”[Mesh] OR “Brown Recluse Spider”[tiab] OR “Brown Recluse”[tiab] OR “Loxosceles reclusa”[tiab] OR “Loxosceles”[tiab] OR “Recluse Spider”[tiab] OR “Spider bite”) AND (“Loxoscelism” OR “Necrotic arachnidism” OR “Spider envenomation”) NOT (“Animal” OR “Veterinary”)
Embase	“loxosceles spider”/exp OR “loxosceles”/exp OR “brown recluse spider”:ti,ab OR “loxosceles reclusa”:ti,ab OR “recluse spider”:ti,ab) AND (“spider bite”/exp OR “loxoscelism”:ti,ab OR “spider envenomation”:ti,ab OR “necrotic arachnidism”:ti,ab) NOT (“animal study”/exp)
Web of Science	(“Brown recluse spider” OR “Loxosceles reclusa” OR “Recluse spider” OR Loxosceles) AND (“Loxoscelism” OR “Spider bite” OR “Spider envenomation” OR “Necrotic arachnidism”) NOT (Animal OR “Veterinary”)

Abbreviations: ab, search abstract; AND, logical “and” operation that returns the article if both operands are true; /exp, an “explosion” in Emtree, so the search looks not only for the subject term you selected but also many related subjects; MeSH, Medical Subject Headings—a comprehensive controlled vocabulary for the purpose of indexing journal articles and books in the life sciences; OR, logical “or” operation that returns an article if either operand is true; ti, search title; tiab, search title and abstract.

For all reports, we compiled the following details for each individual patient: demographic information (age, sex); whether the spider was seen, captured, and identified at the time of or immediately after the arthropod bite; the presence or absence of cutaneous necrosis; whether systemic symptoms occurred (eg, fever, nausea, vomiting, malaise, headache); the presence or absence of hemolytic anemia; coagulation laboratory studies (PT/INR, PTT, fibrinogen, coagulation factor levels, dimerized plasmin fragment D [D-dimer]/fibrin degradation products); the lowest platelet count reported; whether the patient was clinically diagnosed with DIC; and outcome with respect to mortality (ie, alive or dead).

### Statistical analysis

All statistical analyses were performed using Prism, version 10.2.3, software (GraphPad Software). Means (SDs) were reported for normally distributed data, and medians (IQRs) were reported for non-normally distributed data. Differences in means were assessed using 2-sided *t* tests, and differences in medians were assessed using the Mann-Whitney *U* test. Fisher exact test was used to calculate odds ratios (ORs) with 95% CIs. If an individual was reported to be younger than 1 year of age, that individual was considered to be 1 year old for purposes of age-related data analysis. *P* < .05 was considered statistically significant.

## RESULTS

### Case descriptions

#### Case 1

**Figure 1:**
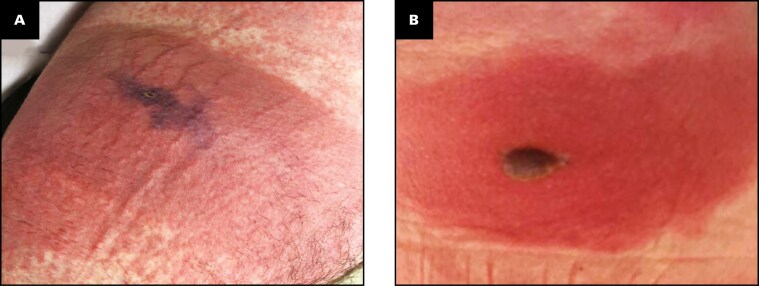
Photographs of the Lesions. **A** Case 1 demonstrates a violaceous, necrotic plaque with central ulceration; **B** Case 2 demonstrates an edematous, erythematous plaque with a central hemorrhagic bulla.

A 29-year-old man presented following a presumed bug bite of unknown etiology on the left flank with subsequent development of a full-body rash. Upon admission, he had fever, leukocytosis, mild anemia, and hematuria. Physical examination revealed a violaceous plaque with visible punctum, surrounded by a well-demarcated erythematous, edematous plaque (Figure 1A), and a morbilliform eruption involving his trunk and extremities. The patient was initially treated empirically with cefepime, linezolid, clindamycin, and doxycycline for possible bacterial infection or tick-borne illness. The toxicology service was consulted for possible cutaneous-hemolytic loxoscelism (Table 2).

**Table 2:** Laboratory Findings in Case 1

Laboratory test	Reference range	Day after envenomation									
		4	5	6	7	8	9	10	11	12	16
White blood cell count, x10^9^/L	3.9-10.7	25.8	26.2	32.4	26.0	21.9	19.4	21.2	18.5	11.8	7.2
Hematocrit, %	41-49	37	36	34	30	29	33	32	32	35	39
Hemoglobin, g/dL (g/L)	14.0-18.1 (140 - 181)	13.1 (131)	12.4 (124)	11.7 (117)	10.1 (101)	10.0 (100)	11.2 (112)	10.5 (105)	10.8 (108)	11.5 (115)	13.0 (130)
Reticulocyte count, %	0.5-2.0	Not performed									
Platelet count, x10^9/L	135-371	231	255	239	171	140	116	98	112	175	368
PT, s	11.9-14.5	14.2	15.3	14.1	14.5	14.6	14.7	14.4	14.9	14.3	Performed twice: 14.1 and 13.9
PTT, s	23.5-33.5	23.3	27.7	24.6	26.4	28.9	39.7	33.3	50.2	55.9	Performed twice: 52.2 and 53.0
Fibrinogen, mg/dL (g/L)	188-450 (1.88-4.50)	—	—	—	583 (5.83)	—	606 (6.06)	—	—	—	—
D-dimer, ng/mL	<500	Not performed									
Lactate dehydrogenase, U/L (ukat/L)	125-220 (2.08-3.67)	290 (4.83)	375 (6.25)	564 (9.40)	646 (10.77)	879 (14.65)	940 (15.67)	947 (15.79)	—	—	—
Haptoglobin, mg/dL (g/L)	14-258 (0.14-2.58)	189 (1.89)	165 (1.65)	61 (0.61)	<8 (<0.08)	<8 (<0.08)	<8 (<0.08)	<8 (<0.08)	10 (0.10)	—	—
C-reactive protein, mg/L	0-5.0	—	—	—	103.6	—	—	—	—	—	—
Total bilirubin, mg/dL (µmol/L)	0.2-1.2 (3.4-20.5)	—	2.6 (44.5)	4.8 (82.1)	5.4 (92.3)	6.0 (102.6)	2.6 (44.5)	1.1 (18.8)	0.9 (15.4)	—	—
Conjugated bilirubin, mg/dL (µmol/L)	0-0.5 (0-8.6)	—	0.8 (13.7)	0.6 (10.3)	0.9 (15.4)	1.1 (18.8)	0.9 (15.4)	0.4 (6.8)	0.3 (5.1)	—	—
Aspartate aminotransferase, U/L (ukat/L)	5-40 (0.08-0.66)	—	17 (0.28)	29 (0.48)	28 (0.46)	34 (0.56)	33 (0.55)	38 (0.63)	29 (0.48)	—	—
Alanine aminotransferase, U/L (ukat/L)	0-55 (0.00-0.91)	—	67 (1.11)	60 (1.00)	47 (0.78)	40 (0.66)	42 (0.70)	55 (0.91)	62 (1.03)	—	—
Alkaline phosphatase, U/L (ukat/L)	40-150 (0.67-2.50)	—	87 (1.45)	71 (1.18)	77 (1.28)	60 (1.00)	67 (1.12)	54 (0.90)	55 (0.92)	—	—
Creatinine kinase, U/L (ukat/L)	30-200 (0.50-3.33)	—	80 (1.33)	75 (1.25)	74 (1.23)	92 (1.53)	84 (1.40)	64 (1.07)	73 (1.22)	—	—
Serum urea nitrogen, mg/dL (mmol/L)	7-21 (2.5-7.5)	11 (3.9)	15 (5.4)	16 (5.7)	19 (6.8)	16 (5.7)	15 (5.4)	12 (4.3)	12 (4.3)	—	—
Urine blood, urinalysis	Negative	Negative	Negative	Negative	Trace	Small	Small	Trace	Negative	Negative	—
Direct antiglobulin test (immunoglobulin G)	Negative	—	—	—	—	—	Negative	—	—	—	—
Direct antiglobulin test (C3)	Negative	—	—	—	—	—	Negative	—	—	—	—

Abbreviations: D-dimer, dimerized plasmin fragment D; PT, prothrombin time; PTT, partial thromboplastin time.

**Figure 2:**
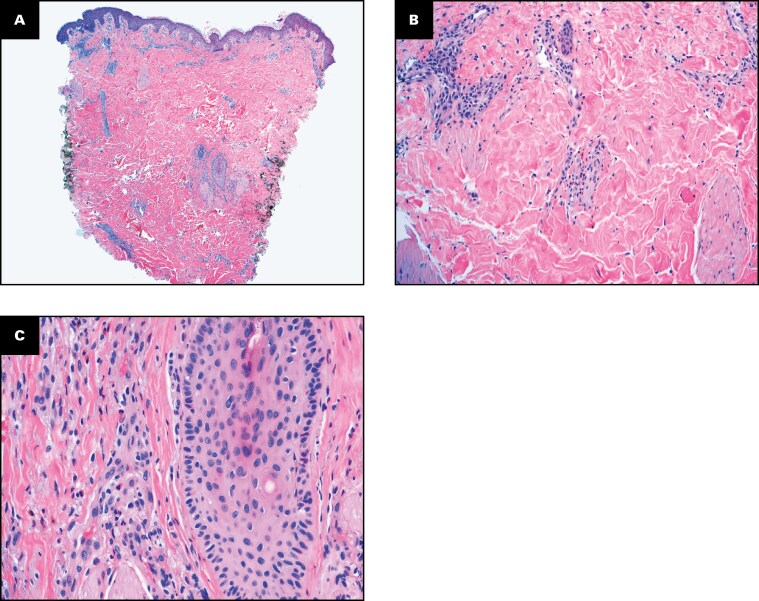
Photographs of the Histopathologic Findings of the Cutaneous Punch Biopsy Obtained From the Patient Described in Case 1 (H&E; **A**, ×40; **B**, ×200; **C**, ×400).

Comprehensive evaluation included negative *Rickettsia* serologies, *Ehrlichia/Anaplasma* nucleic acid polymerase chain reaction, and negative tissue cultures (bacterial, fungal, acid-fast bacilli). Cutaneous punch biopsy demonstrated epidermal spongiosis, papillary dermal edema, and mixed dermal inflammation with numerous eosinophils suggestive of an arthropod or arachnoid bite reaction (Figure 2). Although the patient did not identify a brown recluse spider, given that he resided in the spider’s endemic area, the dermatology and toxicology services felt that the clinical presentation was most consistent with a brown recluse spider bite.

**Figure 3:**
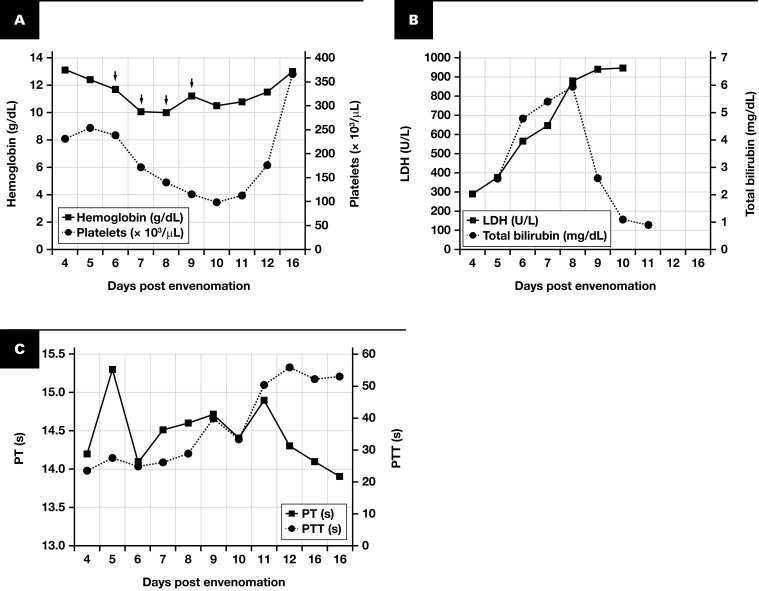
Laboratory Parameters in Case 1. **A** Hemoglobin and platelet counts, **B** lactate dehydrogenase (LDH) and total bilirubin levels, and **C** prothrombin time (PT) and activated partial thromboplastin time (PTT) over the patient’s hospital course. Arrows illustrate days on which at least 1 Red Blood Cell unit was transfused.

Coagulation testing revealed a normal PTT on admission (day 4 after envenomation), with progressively increasing prolongation (Table 2, Table 3; Figure 3). The prolonged PTT on day 16 after envenomation did not correct upon mixing with PNP, but intrinsic pathway coagulation factor activity levels were within normal ranges or elevated, arguing against a specific coagulation factor inhibitor (Table 3). The STA-Staclot LA assay, a test for lupus anticoagulant, was positive, while a second type of lupus anticoagulant test, the dRVVT, was negative. Of note, no anticoagulant therapy (eg, unfractionated heparin, low-molecular-weight heparin, anti-Xa therapy) was administered.

**Table 3:** Coagulation Studies in Case 1 (Day 16 After Envenomation)

Test	Reference range	Result
PT, s	11.9-14.5	14.1
PTT, s	23.5-33.5	52.2
Thrombin time, s	15.0-25.0	16
PTT mixing study, s		
PNP (0 h)	23.5-33.5	28.0
0 h (patient + PNP)	23.5-33.5	42.9
1 h incubation at 37 °C (patient + PNP)	23.5-33.5	45.9
2 h incubation at 37 °C (patient + PNP)	23.5-33.5	48.5
STA-Staclot LA assay	Negative (<8 s)	Positive (35.2 s)
dRVVT		
Screen	0.7-1.2	Positive (1.5)
1:1 mix	—	Negative (1.1)
Confirmation	—	Not performed
Factor II, %	50-150	127
Factor V, %	50-150	117
Factor VIII, %	50-150	141
Factor IX, %	50-150	181
Factor X, %	50-150	118
Factor XI, %	50-150	142

Abbreviations: dRVVT, dilute Russell viper venom time; PNP, pooled normal plasma; PT, prothrombin time; PTT, partial thromboplastin time.

The hospital course was complicated by systemic inflammatory response syndrome, hemolytic anemia, and thrombocytopenia. The patient was switched to vancomycin and ceftriaxone for presumed cellulitis and received 9 units of red blood cells (4580 mL) over the course of 4 days (days 6-9 after envenomation) for hemolytic anemia. There were likely components of both intravascular and extravascular hemolysis given the laboratory findings of undetectable haptoglobin, spherocytosis on peripheral blood smear, and increased urine urobilinogen and urine blood without red blood cells. Plasma-free hemoglobin was not assessed. The patient’s hemoglobin and hematocrit nadir were 9.1 g/dL (91 g/L) and 26%, respectively. The large quantity of blood products transfused was because our hospital employs an aggressive blood transfusion strategy for patients with loxoscelism, given that the hemolysis can be precipitous and severe. Generally, a hematocrit threshold of 30% is used in otherwise healthy adults without baseline anemia. As the patient remained relatively hemodynamically stable and manageable with blood product transfusion alone, additional interventions, such as complement inhibitors (eg, eculizumab) or therapeutic plasma exchange, were not considered. If he had decompensated, however, these approaches may have been employed.

Upon discharge, the patient’s hematocrit level was stable, total bilirubin level was within normal limits, and urinalysis was without blood. There was no definitive clinical evidence of hemorrhage or DIC throughout the hospital course. The only laboratory abnormality at discharge was a persistently prolonged PTT. Repeat laboratory testing (PT, PTT, complete blood cell count) following discharge was ordered, but the patient failed to return for evaluation and was lost to follow-up.

#### Case 2

**Figure 4:**
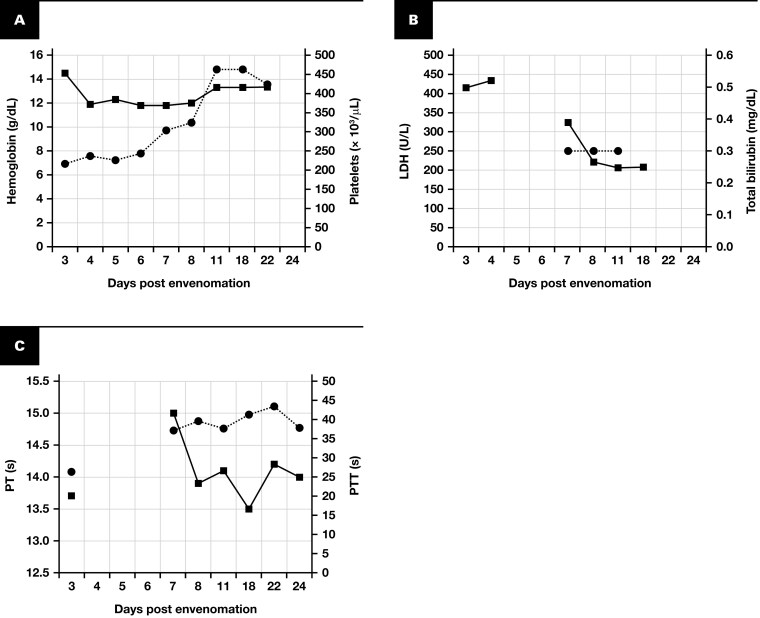
Laboratory Parameters in Case 2. **A** Hemoglobin and platelet counts, **B** lactate dehydrogenase (LDH) and total bilirubin levels, and **C** prothrombin time (PT) and activated partial thromboplastin time (PTT) over the patient’s hospital course.

A 71-year-old woman with a history of arthritis, chronic obstructive pulmonary disease, papillary thyroid carcinoma, and hematuria presented with worsening malaise, nausea, vomiting, shortness of breath, fever, diaphoresis, and a new skin lesion on her right flank. The patient did not recall an arthropod bite. On physical examination, a well-demarcated, edematous, erythematous plaque with a central hemorrhagic bulla was noted on the right flank (Figure 1B). Over the next several days, the right flank lesion ulcerated, and she developed edematous, erythematous papules and plaques of the right chest, back, and lower extremities. The toxicology and dermatology services were consulted. Although the patient did not identify a brown recluse spider, given that she resided in the spider’s endemic area, both dermatology and toxicology felt that the clinical presentation was most consistent with a brown recluse spider bite. Laboratory evaluation suggested the possibility of hemolysis (Table 4; Figure 4).

**Table 4:** Laboratory Findings in Case 2

Laboratory test	Reference range	Days after envenomation									
		3	4	5	6	7	8	11	18	22	24
White blood cell count, x10^9^/L	3.9-10.7	11.8	10.7	11.4	10.6	10.9	8.7	10.3	7.8	7.3	—
Hematocrit, %	36-43	42	36	36	36	35	36	41	41	40	—
Hemoglobin, g/dL (g/L)	11.8-16.0 (118-160)	14.5 (145)	11.9 (119)	12.3 (123)	11.8 (118)	11.8 (118)	12.0 (120)	13.3 (133)	13.3 (133)	13.4 (134)	—
Reticulocyte count, %	0.5-2.0	Not performed									
Platelet count, x10^9^/L	135-371	215	237	224	242	302	322	463	462	424	—
PT, s	11.9-14.5	13.7	—	—	—	15.0	13.9	14.1	13.5	14.2	14.0
PTT, s	23.5-33.5	26.2	—	—	—	37.1	39.6	37.5	41.3	43.2	37.6
Fibrinogen, mg/dL (g/L)	188-450 (1.88-4.50)	Not performed									
D-dimer, ng/mL	<500	Not performed									
Lactate dehydrogenase, U/L (ukat/L)	125-220 (2.08-3.67)	415 (6.92)	433 (7.22)	—	—	325 (5.42)	221 (3.68)	206 (3.43)	208 (3.47)	—	—
Haptoglobin, mg/dL (g/L)	40-273 (0.40-2.73)	295 (2.95)	284 (2.84)	—	—	398 (3.98)	415 (4.15)	392 (3.92)	306 (3.06)	—	—
C-reactive protein, mg/L	0-5.0	Not performed									
Total bilirubin, mg/dL (µmol/L)	0.2-1.2 (3.4-20.5)	0.5 (8.55)	—	—	—	0.3 (5.13)	0.3 (5.13)	0.3 (5.13)	—	—	—
Conjugated bilirubin, mg/dL (µmol/L)	0-0.5 (0-8.6)	0.2 (3.42)	—	—	—	0.1 (1.71)	0.1 (1.71)	—	—	—	—
Aspartate aminotransferase, U/L (ukat/L)	5-40 (0.08-0.66)	41 (0.68)	—	—	—	47 (0.78)	38 (0.63)	27 (0.45)	—	—	—
Alanine aminotransferase, U/L (ukat/L)	0-55 (0.00-0.91)	58 0.96)	—	—	—	54 (0.90)	54 (0.90)	37 (0.61)	—	—	—
Alkaline phosphatase, U/L (ukat/L)	40-150 (0.67-2.50)	104 (1.73)	—	—	—	151 (2.52)	149 (2.48)	114 (1.90)	—	—	—
Creatinine kinase, U/L (ukat/L)	29-168 (0.48-2.80)	143 (2.38)	85 (1.42)	—	—	30 (0.50)	21 (0.35)	30 (0.50)	—	—	—
Serum urea nitrogen, mg/dL (mmol/L)	8-26 (2.86-9.28)	22 (7.86)	13 (4.64)	10 (3.57)	14 (5.00)	18 (6.43)	14 (5.00)	12 (4.29)	—	—	—
Urine blood (urinalysis)	—	Moderate	—	—	—	—	Negative	—	—	—	—
Direct antiglobulin test	Negative	Not performed									

Abbreviations: D-dimer, dimerized plasmin fragment D; PT, prothrombin time; PTT, partial thromboplastin time.

Coagulation studies revealed a normal PTT on admission (presumed day 3 after envenomation) that became prolonged during the patient’s hospital stay. On day 24 after envenomation, testing demonstrated a prolonged PTT that corrected to normal upon mixing with PNP. Coagulation factor activity levels for intrinsic pathway proteins were within the normal range or slightly elevated (Table 5). The STA-Staclot LA and dRVVT tests for lupus anticoagulant were negative.

**Table 5:** Coagulation Studies in Case 2 (Day 24 After Envenomation)

Test	Reference range	Result
PT, s	11.9-14.5	14.0
PTT, s	23.5-33.5	37.6
Thrombin time, s	15.0-25.0	19
PTT mixing study, s		
PNP	23.5-33.5	28.4
0 h (patient + PNP)	23.5-33.5	29.5
1 h incubation at 37 °C (patient + PNP)	23.5-33.5	30.9
2 h incubation at 37 °C (patient + PNP)	23.5-33.5	31.6
STA-Staclot LA assay	Negative (<8 s)	Negative
dRVVT		
Screen	0.7-1.2	Positive (1.3)
1:1 mix	—	Negative (1.1)
Confirmation	—	Not performed
Factor II, %	50-150	149
Factor V, %	50-150	127
Factor VIII, %	50-150	212
Factor IX, %	50-150	181
Factor X, %	50-150	126
Factor XI, %	50-150	142

Abbreviations: dRVVT, dilute Russell viper venom time; PNP, pooled normal plasma; PT, prothrombin time; PTT, partial thromboplastin time.

No clinical evidence of hemolytic anemia, hemorrhage, or coagulopathy was observed during hospitalization, and blood product administration was not required. The patient’s condition was managed with supportive care only. No anticoagulant therapy was administered. Upon discharge, the prolonged PTT was the most notable laboratory abnormality.

### Literature review

**Figure 5:**
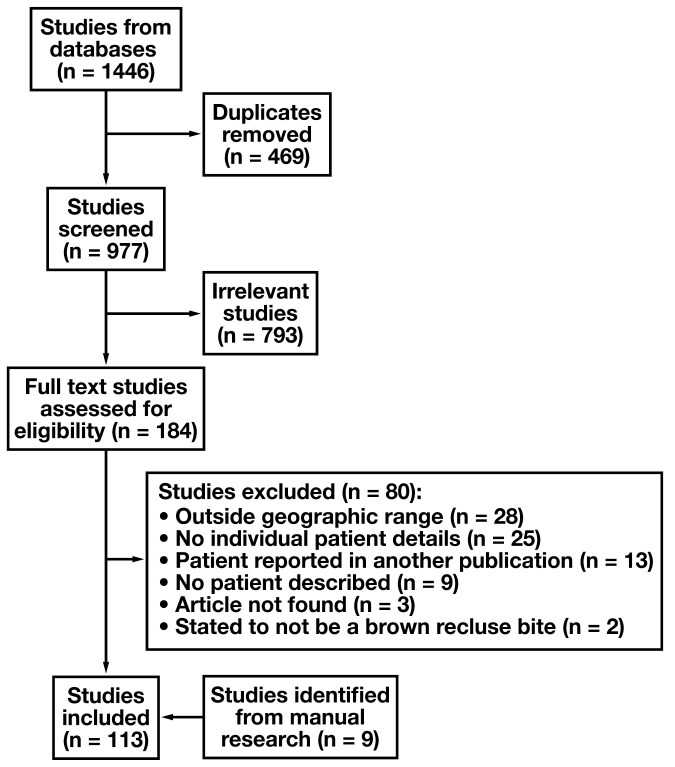
Flow Diagram of Literature Search Results.

Following removal of duplicate reports, a total of 977 studies were screened, 793 of which were deemed irrelevant based on their title and abstract (Figure 5). The 184 remaining full-text studies were assessed for eligibility. Eighty were excluded, primarily because reported cases of *L reclusa* envenomation occurred outside the accepted endemic region for *L reclusa* (n = 28) or because the study did not include individual patient details (n = 25). Other reasons for exclusion were that the patient was described in a separate publication that was already included in the review (n = 13) or the article did not report a patient (n = 9) or specifically stated that a patient did not or was unlikely to have a brown recluse bite (n = 2). Finally, 3 articles could not be located online or with the assistance of a biomedical librarian. Nine additional studies were included following manual search of the reference lists of the included articles.

The 113 articles included in this study described 263 individuals with reported *L reclusa* envenomation (Supplementary Table S1). Age was reported for 245 individuals, with a median age of 23 years (IQR, 12-40 years) (Table 6). Sex was reported for 249 individuals (125 female and 124 male). There was no difference in the median age of female and male individuals (23.5 years [IQR, 14-36 years] vs 23 years [IQR, 11-42.5 years]; *P* = .77). Forty-six patients had observed a spider at the time or immediately following envenomation. Of these, 17 patients reported that the spider was identified as *L reclusa* by a physician. Only 4 patients reported that an expert (toxicologist, arachnologist, or entomologist) confirmed the spider as *L reclusa*. The most common anatomic location of the bite was the lower extremity (97/263 [36.9%]—ankle, foot, leg, thigh). Cutaneous necrosis or eschar formation at the bite was reported in 51.7% (136/263) of cases. Subjective or objective findings of systemic loxoscelism occurred in 57.0% (150/263) of cases, and hemolysis occurred in 41.8% (110/263) of cases.

**Table 6:** Details of the 263 Cases of *Loxosceles reclusa* Envenomation Identified in the Literature

Characteristics	Value
Age, median (IQR), y (n = 245)	23 (12-40)
Age group, No. (%), y	
<1	4 (1.5)
1-5	14 (5.3)
6-10	35 (13.3)
11-15	26 (9.9)
16-20	34 (12.9)
21-30	43 (16.3)
31-40	31 (11.8)
41-50	33 (12.5)
51-60	13 (4.9)
61-70	6 (2.3)
>70	6 (2.3)
Unknown/NR	18 (6.8)
Sex, No. (%)	
Female	125 (47.5)
Male	124 (47.1)
Unknown/NR	14 (5.3)
Spider seen, captured, or identified, No. (%)	
Patient observed spider	
Yes	46 (17.5)
No	33 (12.5)
Unknown/NR	184 (70.0)
Spider identified by physician	
Yes	17 (6.5)
No	80 (30.4)
Unknown/NR	166 (63.1)
Spider identified by expert^a^	
Yes	4 (1.5)
No	90 (34.2)
Unknown/NR	169 64.3)
Anatomic location of bite,^b^ No. (%)	
Lower extremity	97/263 (36.9)
Torso	57/263 (21.7)
Upper extremity	56/263 (21.3)
Head/neck	27/263 (10.3)
Unknown/NR	26/263 (9.9)
Cutaneous necrosis or eschar, No. (%)	
Yes	136 (51.7)
No	79 (30.0)
Unknown/NR	48 (18.3)
Systemic signs or symptoms,^c^ No. (%)	
Yes	150 (57.0)
No	91 (34.6)
Unknown/NR	22 (8.4)
Hemolysis, No. (%)	
Yes	110 (41.8)
No	130 (49.4)
Unknown/NR	23 (8.7)
DIC probable or likely,^d^ No. (%)	
Yes	12 (4.6)
No	234 (89.0)
Unknown/NR	17 (6.5)
Mortality, No. (%)	
Alive	253 (96.2)
Deceased	10 (3.8)
Unknown/NR	0 (0.0)

Abbreviations: DIC, disseminated intravascular coagulation; NR, not reported.

^a^Toxicologist, arachnologist, or entomologist.

^b^Lower extremity (ankle, foot, leg, thigh); torso (abdomen, axilla, back, breast, buttocks, chest, flank, genitalia); upper extremity (arm, finger, hand, shoulder); head/neck (face, head, neck).

^c^At least 1 of fever, chills, headache, malaise, nausea, vomiting, hemolysis, coagulopathy, or hypotension.

^d^As reported by the authors.

At least 1 coagulation laboratory result was reported in 54 cases, at least 1 platelet count was reported in 75 cases, and 43 cases reported at least 1 of each (ie, both a coagulation laboratory parameter and a platelet count) (Table 7). Most of the cases that included coagulation laboratory findings included only a qualitative result (ie, normal or abnormal) at 1 time point. Even among those cases that included a quantitative result, the majority did not include a reference range, precluding interpretation of the laboratory values. Among cases that included a reference range or reported a qualitative interpretation, 22 of 54 cases had normal clotting times (PT/INR or PTT), while 12 of 54 had at least 1 clotting time that was reportedly prolonged. Nineteen cases reported at least 1 qualitative or quantitative fibrinogen result, with a median value of 400 mg/dL (4.00 g/L) (IQR, 193.5-550.0 mg/dL; 1.94-5.50 g/L). Only 7 provided a qualitative interpretation or reference range, among which the fibrinogen level was decreased in 2 cases, normal in 2 cases, and elevated in 3 cases. Analysis of other coagulation factors was reported in 1 case in which factor VIII was decreased at 6%, with a specific inhibitor (1.25 Bethesda units), while levels of factors II, V, VII, XI, X, and XI were normal, and results of lupus anticoagulant testing were negative.^31^

**Table 7:** Details of the 86 Cases With Coagulation Laboratory or Platelet Count Results Reported

Age, y	Sex	Bite location	Necrosis/eschar?^a^	Hemolysis?^a^	Details of coagulation laboratory results^a^^,^^b^^,^^c^	Lowest platelet count reported, ×10^9^/L	DIC?^a^
9	M	Torso	Yes	Unknown	NR	645	No
7	M	Lower extremity	No	No	Performed on day +1: PT, 10.4 s; PTT, 28.0 s (both normal); fibrinogen, 558 mg/dL (5.58 g/L) (elevated)	282	No
24	M	Torso	No	Yes	NR	184	No
59	F	Upper extremity	Unknown	No	PT, PTT, normal; ristocetin cofactor, 339%; von Willebrand factor antigen, 306%; von Willebrand factor to factor VIII binding capacity, normal; platelet functional assay, normal; factor levels for II, V, VII, XI, X, and XI as well as lupus anticoagulant, within normal range; Bethesda assay showed a low titer of antibody against factor VIII (1.25 Bethesda unit); factor VIII low at 6%.	Normal	No
49	M	NR	Yes	Yes	NR	112	No
11	M	Head/neck	Yes	Yes	Normal	NR	No
19	M	Lower extremity	Yes	No	NR	96	No
3	F	Torso	Unknown	Yes	PT, 19.8 s; INR, 1.8; PTT, 40.2 s	54	Yes
19	M	Upper extremity	No	Yes	INR normal	Normal	No
27	M	Lower extremity	Yes	No	Normal	Normal	No
10	F	Head/neck	Yes	Yes	PT, 12.5 s; PTT, 21.1 s; fibrinogen, 542 mg/dL (5.42 g/L); D-dimer, positive	235	No
46	M	Lower extremity	No	Yes	PT, 15 s	NR	No
20	M	Lower extremity	Yes	Yes	INR, 1.55	264	No
20	F	Head/neck	No	No	PT, 14.2 s (elevated); PTT, normal	NR	No
14	F	Torso	No	No	Normal	241	No
6	M	Torso	Yes	Yes	PTT, 77.4 s (reference range: <50 s); PT, 21.9 s (reference range: 12.9 s); fibrinogen, 88 mg/dL (0.88 g/L) (reference range: >160 mg/dL, [>1.60 g/L])	25	Yes
44	M	Head/neck	Unknown	No	Fibrin degradation products, >20 µg/mL; fibrinogen, 501 mg/dL (5.01 g/L); D-dimer, 10.8 µg/mL; INR, 2.0; PTT, 62.6 s	99	Yes
15	F	NR	No	Yes	Normal	Normal	No
16	M	Torso	Yes	Yes	PT, 15.1 s (reference range: 9.3-11.3 s); PTT, 51.5 s (reference range: 23.0-34.0 s)	203	No
24	F	Toros	Yes	Yes	INR and PTT, mildly elevated; D-dimer and fibrinogen, elevated	100	No
6	M	Upper extremity	No	Yes	PTT, 34 s (reference range: 25-40 s); PT, 12 s (reference range: 10-13 s)	240	No
12	F	Lower extremity	No	Yes	Normal	145	No
44	M	Torso	Yes	Yes	NR	179	No
4	F	Head/neck	Yes	Yes	NR	176	No
31	F	Head/neck	No	No	Normal	Normal	No
32	F	Upper extremity	Yes	Yes	PT, 12.1 s (normal); PTT, 28.0 s (normal); fibrinogen, 389 mg/dL (3.89 g/L); D-dimer, 955 ng/mL (reference range: <500 ng/mL)	110	No
41	F	Upper extremity	Yes	Yes	Normal PT, PTT, and INR	Normal	No
7	F	Torso	No	Yes	Prolonged PT and PTT	NR	Yes
17	F	Lower extremity	Yes	Yes	INR, 1.8	NR	No
12	F	Torso	Unknown	Yes	Normal	Normal	No
NR	NR	NR	Unknown	Yes	NR	136	No
NR	NR	NR	Unknown	Yes	NR	365	No
NR	NR	NR	Unknown	Yes	NR	152	No
NR	NR	NR	Unknown	Yes	NR	179	No
NR	NR	NR	Unknown	Yes	NR	136	No
NR	NR	NR	Unknown	Yes	NR	124	No
NR	NR	NR	Unknown	Yes	NR	117	No
NR	NR	NR	Unknown	Yes	NR	144	No
NR	NR	NR	Unknown	Yes	NR	320	No
32	F	Torso	No	No	Normal	Normal	No
27	F	Upper extremity	No	Yes	Fibrin split products normal	Normal	No
34	F	Lower extremity	No	No	NR	Normal	No
NR	F	Lower extremity	No	No	NR	Normal	No
37	F	Torso	Yes	Yes	PT, 13.5 s (reference range: 10.2-13.0 s); INR, 1.1 (reference range: 0.9-1.3); PTT, 37.3 s (reference range: 25.1-41.5 s); fibrinogen, 760 mg/dL (7.60 g/L)	140	No
7	M	Head/neck	Yes	No	Normal	Normal	No
16	M	Upper extremity	Yes	Yes	PTT, 40.7 s (high); INR, 3 (high); D-dimer, 1804 ng/mL; fibrinogen, 217 mg/dL (2.17 g/L) (normal)	72	Yes
19	M	Lower extremity	Yes	Yes	PT, 13 s (reference range: 9-12 s)	53	No
22	F	Upper extremity	Yes	Yes	INR, 1.35; fibrinogen, 384 mg/dL (3.84 g/L)	194	No
10	F	Torso	Yes	Yes	INR, 2.0 s; PTT, 37.5 s	78	Yes
25	M	Lower extremity	Yes	Yes	PT, 12.1 s (reference range: 9.5-14.2 s); PTT, 29.9 s (reference range: 24.0-36.5 s); INR, 1.1; fibrinogen, 702 mg/dL (7.02 g/L); D-dimer 1611 ng/mL (reference range: <500 ng/mL)	174	No
22	F	Upper extremity	No	Yes	NR	130	No
70	M	Lower extremity	Yes	No	PT, 44.3 s (otherwise normal)	Normal	No
26	F	Upper extremity	Yes	Yes	PT, >20 s; PTT, >100 s; fibrinogen, <50 mg/dL (<0.50 g/L)	143	Probable
17	F	Upper extremity	No	Yes	Normal PT and PTT; fibrinogen, 402 mg/dL (4.02 g/L)	210	No
9	F	Torso	Yes	Yes	Mildly prolonged INR	NR	No
8	F	Torso	No	Yes	PT, 24.8 s; PTT, 47 s; fibrinogen, 278 mg/dL (2.78 g/L)	20	Probable
13	M	Torso	Yes	Yes	INR, 1.9 (reference range: 0.9-1.2); PTT, 39 s (reference range: 26-37 s); D-dimer, 5781 mg/mL	100	No
19	M	Torso	No	No	NR	176	No
16	M	Torso	No	Yes	Normal	166	No
15	M	Upper extremity	No	Yes	PT, 16.2 s	NR	No
15	F	Lower extremity	Unknown	Yes	PT, 21.1 s	NR	No
12	M	Lower extremity	Yes	Yes	PT, 13.9 s	NR	No
10	M	Torso	Yes	Yes	PT, 13.8 s	NR	No
11	M	Upper extremity	No	Yes	PT, 13.0 s	NR	No
6	M	Torso	No	Yes	PT, 30.1 s; PTT, 65.6 s; INR, 2.96; D-dimer, 12 572 ng/mL; fibrinogen, 164 mg/dL (1.64 g/L)	121	Yes
30	M	Upper extremity	Yes	Yes	NR	134 (reference range: 130-400)	No
28	F	Torso	No	Yes	PT, 10.9 s; PTT, 28 s; fibrinogen, >860 mg/dL (>8.60 g/L)	251	No
34	F	Upper extremity	Yes	Yes	INR, 1.51; PTT, 28.1 s; fibrinogen, 400 mg/dL (4.00 g/L)	292	No
NR	M	Lower extremity	Yes	Yes	NR	145	No
9	F	Head/neck	Yes	Yes	PT, 12.4 s (normal); PTT, 29.9 s (normal); fibrinogen, 410 mg/dL (4.10 g/L) (elevated)	70	No
19	M	Torso	Yes	No	NR	Normal	No
42	M	Lower extremity	No	No	Normal PT and PTT	186	No
26	F	Torso	Yes	Yes	NR	168	No
5	F	Torso	Unknown	Yes	NR	169	No
<1	F	Head/neck	No	Yes	NR	357	No
23	M	Lower extremity	Yes	No	NR	Normal	No
8	M	Torso	Yes	Yes	NR	67	No
20	M	Torso	No	No	NR	Normal	No
5	M	Lower extremity	Yes	Yes	PT, 10.9 s; PTT, 40.3 s; fibrinogen, 170-185 mg/dL (1.7-1.85 g/L)	100	Yes
4	F	Head/neck	No	Yes	NR	176	No
2	M	NR	No	Yes	NR	150	No
10	M	Upper extremity	Yes	Yes	NR	54	No
25	F	Upper extremity	Yes	Yes	PT, 17.8 s	68	No
6	M	Torso	Yes	Yes	Normal	75	No
65	M	Torso	Yes	Yes	NR	50	Possible
23	F	Lower extremity	Yes	Yes	Normal fibrinogen and PTT	Normal	No

Abbreviations: D-dimer, dimerized plasmin fragment D; DIC, disseminated intravascular coagulation; F, female; INR, international normalized ratio; M, male; NR, not reported; PT, prothrombin time; PTT, partial thromboplastin time.

^a^As reported by the authors.

^b^Inclusive of any of the following: PT, INR, PTT, fibrinogen, coagulation factor levels, lupus anticoagulant testing, D-dimer, fibrin degradation products/fibrin split products.

^c^Any interpretation of results (ie, normal, abnormal, elevated) is what was reported by the authors; reference range was not reported, except where included.

Among the 75 cases with platelet count results, 58 reported a quantitative value, while 17 reported that the count was “normal.” Among the 58 cases, the median nadir platelet count was 144.5 × 10^9^/L (IQR, 99.75-188 × 10^9^/L). Most cases did not report a reference range, again precluding interpretation of results, but 17 cases reported that the lowest platelet count was between 100 and 150 × 10^9^/L; in 12 cases, the lowest reported result was between 50 and 100 × 10^9^/L, and in 2 cases the lowest count was below 50 ×10^9^/L.

In 93.5% (246/263) of cases, the authors disclosed whether there was clinical concern for DIC. Among these 246 cases, there was clinical concern for DIC in 12 (4.9%: 6 female and 6 male individuals) cases; there was no reported concern for DIC in 95.1% (234/246). The criteria used to determine the presence of DIC differed among the cases, however, and included various combinations of prolonged clotting times, decreased fibrinogen, decreased platelet counts, and elevated fibrin degradation products. In some cases, the criteria on which DIC was based were not reported. The median age of patients with concern for DIC was 8.5 years (IQR, 6.0-23.5 years), with a range of 3 to 65 years. Hemolysis was reported in 83.3% (10/12) of cases with concern for DIC. Hemorrhage was reported in 25.0% (3/12) of cases. In 7 cases, fibrinogen levels were reported: 5 had a level above 100 mg/dL (1.00 g/L) (median, 170 mg/dL (1.70 g/L) [IQR, 126-248 mg/dL (1.26-2.48 g/L)] [range, 50-501 mg/dL (0.50-5.01 g/L)]). Ten cases reported a platelet count: 7 were below 100 × 10^9^/L (median, 75 × 10^9^/L [IQR, 75-99.75 × 10^9^/L] [range, 20-143 × 10^9^/L]). Only 0.8% (2/263) of cases reported all of the requisite data needed to calculate an International Society on Thrombosis and Haemostasis (ISTH) DIC score^32,33^; neither had clinical concern for DIC, and the ISTH DIC score was 2 in each case (ie, not suggestive of overt DIC). Among all cases that reported whether there was concern for DIC (n = 246), there were statistically significantly higher odds of dying if there was clinical concern for DIC compared with no concern for DIC (OR, 82.9 [95% CI, 12.6-433.8]; *P* < .001). Compared with patients who experienced hemolysis without DIC (ie, individuals with severe loxoscelism), patients with DIC still had statistically significantly higher odds of dying (OR, 29.3 [95% CI, 4.4-154.8]; *P*  < 0.001). Death occurred in 41.7% (5/12) of cases with concern for DIC, while the overall reported mortality was 3.8% (10/263).

## DISCUSSION

In this study, we present 2 patients with presumed brown recluse spider envenomation and laboratory evidence for a coagulation abnormality. Although both patients demonstrated a progressively prolonged PTT over their hospital course, the etiology of the abnormal clotting times were not definitively elucidated. In case 1, the abnormality was potentially secondary to the development of a lupus anticoagulant or other, nonspecific inhibitor, which could account for the persistent prolongation even long after the initial envenomation. There was no evidence of an inhibitor in the second case; however, it is possible that a nonspecific inhibitor was present at a low level and was diluted out during mixing, or a non-neutralizing inhibitor developed that was not detected on conventional clot-based coagulation factor activity testing. A coagulation factor deficiency or inhibitor directed against a specific coagulation factor was not identified in either case. Despite the persistently prolonged PTT (at 16 days and 24 days after envenomation in case 1 and case 2, respectively), neither patient demonstrated clinical evidence of bleeding or DIC.

The literature review identified 12 cases of brown recluse envenomation with at least 1 prolonged clotting time, but these cases did not test individual coagulation factors or assess for lupus anticoagulant, precluding identification of the underlying cause. The clinical course and severity of our 2 cases were distinct, with the patient in case 1 experiencing marked hemolysis that required multiple blood transfusions, while the patient in case 2 required only supportive care. As such, perturbations in tests used to assess hemostasis may not reflect the severity of envenomation. Conversely, DIC seems to be rare following brown recluse spider envenomation. The small number of published cases with clinical concern for DIC and the heterogeneity in the criteria by which DIC was considered to be present preclude our ability to recommend an algorithm for predicting DIC or poor outcome. Notably, among the 7 cases with clinical concern for DIC that reported fibrinogen levels, in 5 of the cases, the lowest reported fibrinogen was above 100 mg/dL (1.0 g/L) (range, 164-501 mg/dL; 1.64-5.01 g/L). This finding suggests that a normal fibrinogen level should not be used to exclude DIC in the appropriate clinical and laboratory context because the massive inflammatory state likely mediates elevated levels, despite a global consumptive process. In addition, we observed that DIC is more likely to occur in younger patients (ie, ≤18 years of age), and in individuals with other systemic findings (eg, hemolysis). Disseminated intravascular coagulation is also associated with statistically significantly higher mortality, even compared with patients who have hemolysis or other systemic pathology.

Our study is the largest review of coagulation alterations resulting from *L reclusa* envenomation to date. The majority of previous analyses, with the exception of 1 study,^31^ did not report comprehensive coagulation testing in patient cohorts. To address this limitation, we evaluated multiple coagulation laboratory parameters in our patients across multiple time points.

Currently, there is no accepted reproducible or standard laboratory method to diagnose or predict the outcomes of loxoscelism with respect to coagulopathy. Similar to prior studies that have assessed potential risk factors for hemolytic anemia,^14,16,26^ we found that patients with coagulopathy were younger (ie, ≤18 years of age). Most had concomitant hemolysis. There did not appear to be an association with patient sex, although the small number of patients with coagulopathy precluded comprehensive characterization of additional risk factors. Nevertheless, if a pediatric patient presents with concern for brown recluse spider envenomation in the spider’s endemic region, close monitoring for both hemolysis and coagulopathy is warranted. Assessment for coagulopathy/DIC is imperative particularly if hemolysis appears imminent (ie, via lactate dehydrogenase and bilirubin evaluation).^14^

Previous work has suggested that altered cell surface expression of thrombomodulin and endothelial protein C receptor due to sphingomyelinase D within *Loxosceles* spider venom may be a possible mechanism for the development of DIC.^6,24^ The precise mechanism by which aberrations in hemostatic laboratory parameters and clinical sequelae occur, however, is unknown. Further studies to characterize the mechanism may impart both diagnostic and therapeutic potential. In addition, ascertaining the effect of envenomation on coagulation factors and inhibitor development may expand upon prior work that evaluated laboratory predictors of hemolysis and the development of DIC.^14^ It should be noted that in our review, most patients with concern for DIC also experienced overt hemolysis. As such, it may be challenging to parse out the direct venom effect from the hemolysis on coagulopathy. Nevertheless, as only a small subset of all patients with hemolysis developed clinical concern for DIC, the process is likely multifactorial. By understanding the risk factors for development of systemic loxoscelism, including the potential effects of *Loxosceles* venom on hemostasis, there is an opportunity to identify and triage patients with a high risk of mortality and potentially intervene before the onset of severe disease.

This study had limitations. At the time of these cases, our laboratory did not perform the dRVVT confirmation if the dRVVT mix corrected itself. As such, it is theoretically possible that a low-titer lupus anticoagulant may have been present in case 2 and was not detected.^34^ In addition, we did not test factor XII, prekallikrein, or high-molecular-weight kininogen. Nevertheless, both patients had normal PTTs at baseline, likely excluding a congenital contact factor deficiency, as well as the presence of a lupus anticoagulant or other, nonspecific inhibitor before envenomation. We also did not test fibrin degradation products (eg, D-dimer), precluding our ability to calculate an ISTH DIC score. Further, in case 1, C-reactive protein was elevated at day 7 after envenomation, which can cause a false-positive STA-Staclot LA test for lupus anticoagulant,^35^ although the test was not performed until day 16 after envenomation; nevertheless, we cannot definitively exclude an elevated C-reactive protein as a potential cause of the positive STA-Staclot LA finding. In addition, few cases in the literature reported coagulation laboratory parameters, and even fewer provided a clinical interpretation or reference range. The criteria used to determine DIC differed among studies, and in many studies the criteria used were omitted entirely. Finally, this review of published cases likely overestimated the prevalence of hemolysis and systemic loxoscelism due to more severe cases being seen by a medical professional and a publication bias for severe cases.

Despite the limitations and bias toward the most severe cases, hemostatic perturbations appear to be infrequent, and clinical DIC is uncommon following brown recluse spider envenomation. These findings did reveal that the odds of dying following a brown recluse spider bite are statistically significantly greater if DIC develops, even compared with individuals who have severe loxoscelism and hemolytic anemia without DIC. Although DIC is infrequent overall, it is most common in pediatric patients but can develop at any age. Finally, health care professionals should be cognizant that prolonged clotting times may not necessarily reflect a hemorrhagic predisposition in loxoscelism, and laboratory abnormalities should not be empirically treated; rather, the use of hemostatic agents should be implemented only in the appropriate clinical context.

## Supplementary material

Supplementary material is available at *American Journal of Clinical Pathology* online.

aqaf001_suppl_Supplementary_Table_S1
