# Supplementary material for: Coagulation abnormalities following brown recluse spider (Loxosceles reclusa) envenomation: A description of 2 cases and review of the literature
Source: Am J Clin Pathol. 2025 Jan 30;163(6):822–36. doi: 10.1093/ajcp/aqaf001 (PMC12137049; doi:10.1093/ajcp/aqaf001)
Supplement: aqaf001_suppl_Supplementary_Table_S1 [file aqaf001_suppl_supplementary_table_s1.docx]

| **SUPPLEMENTARY TABLE S1** List of all included articles |
| --- |
| Citation |
| Abraham M, Tilzer L, Hoehn KS, et al. Therapeutic plasma exchange for refractory hemolysis after brown recluse spider (*Loxosceles reclusa*) envenomation. *J Med Toxicol*. 2015;11(3):364-7. doi:10.1007/s13181-015-0485-9. |
| Alqam A, Zakhour J, Karam W, et al. Rare loxoscelism-associated IgG Coombs-positive hemolytic anemia treated successfully with systemic corticosteroids. *Cureus*. 2023;15(10):e47424. doi:10.7759/*cureus*.47424. |
| Anderson PC. Loxoscelism threatening pregnancy: Five cases. *Am J Obstet Gynecol*. 1991;165(5 Pt 1):1454-1456. doi:10.1016/0002-9378(91)90389-9. |
| Anderson PC. Treatment of severe loxoscelism. *Mo Med*. 1971;68:609-611, 618. |
| Anderson PC. What's new in loxoscelism—1978: case report. *Mo Med*. 1977;74(9):549-552, 556. |
| Anwar S, Torosyan R, Ginsberg C, et al. Clinicopathological course of acute kidney injury following brown recluse (Loxoscles reclusa) envenomation. *Clin Kidney J*. 2013;6(6):609-12. doi:10.1093/ckj/sft111. |
| Arnold J, Barlotta K. Young woman with thigh wound and rash. *Ann Emerg Med*. 2018;72(5):615-626. doi:10.1016/j.annemergmed.2018.06.005 |
| Arnold RE. Brown recluse spider bites: five cases with a review of the literature. *JACEP*. 1976;5(4):262-264. doi:10.1016/s0361-1124(76)80005-6. |
| Atkins JA, Wingo CW, Sodeman WA, et al. Necrotic arachnidism. *Am J Trop Med Hyg*. 1958;7(2):165-184. doi:10.4269/ajtmh.1958.7.165. |
| Beamon B, Craddock P. Loxoscelism: a rare presentation of a reclusive diagnosis. *Am J Med Sci. 2023;365(S1)*:S1-S430. |
| Blackall DP. *Transfusion* medicine illustrated: intravascular hemolysis with brown recluse spider envenomation. *Transfusion*. 2004;44(11):1543. doi:10.1111/j.0041-1132.2004.04165.x. |
| Broughton G 2nd. Management of the brown recluse spider bite to the glans penis. *Mil Med*. 1996;161(10):627-629. |
| Cain S, Plapp FV, Dasgupta A, et al. Severe complications in a 25-year-old male after brown recluse spider bite treated by therapeutic plasma exchange: A case report and review of other case studies. *J Clin Apher*. 2023;38(4):505-509. doi:10.1002/jca.22045. |
| Calhoun B, Moore A, Dickey A, et al. Systemic loxoscelism induced warm autoimmune hemolytic anemia: Clinical series and review. *Hematology*. 2022;27(1):543-554. doi:10.1080/16078454.2022.2065086. |
| Cambridge CL, Ezzat A, Papadopoulos A, et al. Venomous vignette: hemolytic anemia triggered by an eight-legged culprit in an adolescent. *Chest*. 2024;166(4):A135-A136. |
| Cassidy M, Craig J, Marrs B. Stellate ganglion blocks to treat CRPS and neuropathy caused by brown recluse spider bite. *Neuromodulation*. 2024;27(7):S55. |
| Chinchanikar S, Khalaf Z, Mateescu V. Brown Recluse Spider Bite: A Case Report of Severe Hemolysis and Sepsis. *Am J Clin Pathol. 2022;158(Supplem*ent_1):S114. doi:10.1093/ajcp/aqac126.242. |
| Chu JY, Rush CT, O'Connor DM. Hemolytic anemia following brown spider (*Loxosceles reclusa*) bite. *Clin Toxicol*. 1978;12:531-534. |
| Dandoy C, Grimley M. Secondary hemophagocytic lymphohistiocytosis (HLH) from a presumed brown recluse spider bite. *J Clin Immunol*. 2014;34(5):544-7. doi:10.1007/s10875-014-0036-1. |
| Dare RK, Conner KB, Tan PC, et al. Brown recluse spider bite to the upper lip. *J Ark Med Soc*. 2012;108(10):208-210. |
| Dillaha CJ, Janser GT, Honeycutt WM, et al. North American loxoscelism - Necrotic bite of brown recluse spider. *JAMA*. 1964;188(1). |
| Donepudi SK, Ahmed KA, Stocks RM, et al. Aural involvement in loxoscelism: case report and literature review. Int *J Pediatr* Otorhinolaryngol. 2005;69(11):1559-1661. doi:10.1016/j.ijporl.2005.04.015. |
| Edwards JJ, Anderson RL, Wood JR. Loxoscelism of the eyelids. *Arch Ophthalmol*. 1980;98(11):1997-2000 |
| Eichner ER. Spider bite hemolytic anemia: positive Coombs' test, erythrophagocytosis, and leukoerythroblastic smear. *Am J Clin Pathol.* 1984;81(5):683-687. doi:10.1093/ajcp/81.5.683. |
| Elbahlawan LM, Stidham GL, Bugnitz MC, et al. Severe systemic reaction to *Loxosceles reclusa* spider bites in a pediatric population. *Pediatr Emerg Care*. 2005;21(3):177-180. |
| Erickson T, Hryhorczuk DO, Lipscomb J, Burda A, Greenberg B. Brown recluse spider bites in an urban wilderness. *J Wilderness Med*. 1990;1(4):258-264. |
| Gehrie EA, Nian H, Young PP. Brown Recluse spider bite mediated hemolysis: clinical features, a possible role for complement inhibitor therapy, and reduced RBC surface glycophorin A as a potential biomarker of venom exposure. *PLoS One*. 2013;8(9):e76558. doi:10.1371/journal.pone.0076558. |
| Ginsburg CM, Weinberg AG. Hemolytic anemia and multiorgan failure associated with localized cutaneous lesion. *J Pediatr*. 1988;112(3):496-499. doi:10.1016/s0022-3476(88)80348-2. |
| Goto CS, Abramo TJ, Ginsburg CM. Upper airway obstruction caused by brown recluse spider envenomization of the neck. *Am J Emerg Med. 1996*;14(7):660-662. |
| Gross AS, Wilson DC, King LE Jr. Persistent segmental cutaneous anesthesia after a brown recluse spider bite. *South Med J*. 1990;83(11):1321-1323. doi:10.1097/00007611-199011000-00025. |
| Hallak A, Mohanakrishnan BPE, Dharmarpandi J, et al. Hold the chemo! Leukostasis, a presentation of brown recluse spider bite: A case report. *J Investig Med High Impact Case Rep*. 2021;9:23247096211039949. doi:10.1177/23247096211039949. |
| Harry S, Brugioni E, Madhusudhana S. Acute hemolytic anemia caused by loxoscelism treated with plasmapheresis: A case report. *J Med Cases*. 2022;13(5):219-224. doi:10.14740/jmc3828. |
| Hassan A, Galipp KM, Sebastian R, Khan O, Sagdeo K. Loxoscelism: Unlocking the bite of hemolysis. *J Investig Med*. 2020;68(2):560. |
| Herman TE, McAlister WH. Epiglottic enlargement: Two unusual causes. *Pediatr Radiol*. 1991;21(2):139-140. doi:10.1007/BF02015631. |
| Hijano DR, Otterson D, Homsi MR, et al. Brown recluse spider bites in patients with neutropenia: A single-institution experience. *J Pediatr* Hematol Oncol. 2019;41(1):28-33. doi:10.1097/MPH.0000000000001253. |
| Hoover EL, Williams W, Koger L, et al. Pseudoepitheliomatous hyperplasia and pyoderma gangrenosum after a brown recluse spider bite. *South Med J*. 1990;83(2):243-6. doi:10.1097/00007611-199002000-00029. |
| Hostetler MA, Dribben W, Wilson DB, et al. Sudden unexplained hemolysis occurring in an infant due to presumed Loxosceles envenomation. *J Emerg Med*. 2003;25(3):277-282. doi:10.1016/s0736-4679(03)00202-6. |
| Ibrahim D, Kao LW, Furbee RB. Credible brown recluse spider bite followed by id reaction and complete recovery. *Clin Toxicol*. 2006;44(5):645-646. |
| Jarvis RM, Neufeld MV, Westfall CT. Brown recluse spider bite to the eyelid. Ophthalmology. 2000;107(8):1492-6. doi:10.1016/s0161-6420(00)00183-4. |
| Khawaja S, Mehaffey R, Calamur N. Secondary HLH – An uncommon manifestation of systemic loxoscelism. *J Hosp Med*. 2023;18: S827-8. |
| Knapp JF, Thomas KR, Mathews R, et al. A 10-year-old female with fever, jaundice, and orthostatic hypotension. *Pediatr Emerg Care*. 1994;10(6):364-368. |
| Kopec G, Wathen D, King M, et al. Being covered in spots is a pain in the gut. *Clin Toxicol*. 2015;53(7):746. |
| Lane DR, Youse JS. Coombs-positive hemolytic anemia secondary to brown recluse spider bite: a review of the literature and discussion of treatment. *Cutis*. 2004;74(6):341-347. |
| Lane L, McCoppin HH, Dyer J. Acute generalized exanthematous pustulosis and Coombs-positive hemolytic anemia in a child following *Loxosceles reclusa* envenomation. *Pediatr Dermatol*. 2011;28(6):685-688. doi:10.1111/j.1525-1470.2010.01302.x. |
| Langner TR, Ganatra HA, Schwerdtfager J, et al. Viscerocutaneous loxoscelism manifesting with myocarditis: A case report. *Am J Case Rep*. 2021;22:e932378. doi:10.12659/AJCR.932378. |
| Laxton SJ, Whetstone D. *Loxosceles reclusa* envenomation causing acute hemolytic anemia: a case report on loxoscelism. *Cureus*. 2024;16(7):e64413. doi:10.7759/*cureus*.64413. |
| Leach J, Bassichis B, Itani K. Brown recluse spider bites to the head: three cases and a review. *Ear Nose Throat J*. 2004;83(7):465-470. |
| Leung LK, Davis R. Life-threatening hemolysis following a brown recluse spider bite. *J Tenn Med Assoc*. 1995;88(10):396-397. |
| Liss D, McDowell C, Dribben W. Cardiac toxicity secondary to *Loxosceles reclusa* envenomation. *J Med Toxicol*. 2019;15(2):84. |
| Losher D. A brown recluse spider bite. *Dermatology Nursing*. 2009;21(6). |
| Lung JM, Mallory SB. A child with spider bite and glomerulonephritis: a diagnostic challenge. *Int J Dermatol*. 2000;39:287-289. |
| Madion DC, Marshall MK, Jenkins CD, Kushner GM. Brown recluse spider bite to the face. *J Oral Maxillofac Surg. 2005;63(12):1774*-8. doi: 10.1016/j.joms.2005.08.012. |
| Madrigal GC, Ercolani RL, Wenzl JE. Toxicity from a bite of the brown spider (Loxosceles reclusus). Skin necrosis, hemolytic anemia, and hemoglobinuria in a nine-year-old child. *Clin Pediatr (Phila). 1972;11(11):641-4.* doi:10.1177/000992287201101113. |
| Magrina JF, Masterson BJ. *Loxosceles reclusa* spider bite: a consideration in the differential diagnosis of chronic, nonmalignant ulcers of the vulva. *Am J Obstet Gynecol*. 1981;140(3):341-3. doi:10.1016/0002-9378(81)90287-8. |
| Mai D, Muthukumarasamy N, Ford B, et al. Systemic loxoscelism leading to autoimmune haemolytic anaemia in a healthy young adult. *BMJ Case Rep*. 2024;17(7):e260045. doi:10.1136/bcr-2024-260045. |
| Major T. Clinical experience with management of 20 brown recluse spider bites: An effective treatment regimen. *Mo Med*. 2017;114(4):258-259. |
| Mani S, Katzman C, Liu V. Histopathology aiding diagnosis of viscerocutaneous loxoscelism in a nonendemic region. *JAAD Case Rep*. 2024;45:11-17. doi:10.1016/j.jdcr.2023.12.014. |
| Marcinko DE, Rappaport MJ. Cutaneous necrotic arachnidism. A case report. *J Am Podiatr Med Assoc*. 1986;76(2):105-8. doi:10.7547/87507315-76-2-105. |
| Masters EJ. A clinical observation: loxoscelism pain managed with lidocaine patch. *South Med J*. 2008;101(5):565-566. doi:10.1097/SMJ.0b013e31816c00d3. |
| Masters EJ. Images in clinical medicine. Loxoscelism. *N Engl J Med.* 1998;339(6):379. doi:10.1056/NEJM199808063390605. |
| McDade J, Aygun B, Ware RE. Brown recluse spider (*Loxosceles reclusa*) envenomation leading to acute hemolytic anemia in six adolescents. *J Pediatr*. 2010;156(1):155-157. doi:10.1016/j.jpeds.2009.07.021. |
| Meadows J, Shayesteh N, Crandall E, et al. Eye-popping spider bite: viscerocutaneous Loxosceles envenomation with orbital compartment syndrome. *Clin Toxicol*. 2023;61. |
| Mehr J, Kim J. The use of therapeutic plasma exchange in systemic loxoscelism induced treatment resistant hemolytic anemia: A case report. *Transfus Apher Sci.* 2024;63(4):103960. doi:10.1016/j.transci.2024.103960. |
| Merrill DR, Long B. Arthropod Assault: a case report of brown recluse envenomation in a training environment. *Mil Med*. 2020 Sep 18;185(9-10):e1880-e1881. doi:10.1093/milmed/usaa092. |
| Michaud ME Gibler WB. Hemolytic anemia and hemoglobinuria due to systemic loxoscelism: report of a case. *J Wilderness Med*. 1991;2(1):49-54. doi:10.1580/0953-9859-2.1.49. |
| Minton SA, Olson C. A case of spider bite with severe hemolytic reaction. Pediatrics. 1964;33:283-4. |
| Mueller M, Doucette E, Freeman S, et al. Viscerocutaneous loxoscelism in an adult with acute generalized exanthematous pustulosis. *Mo Med*. 2014;111(2):139-142. |
| Murray LM, Seger DL. Hemolytic anemia following a presumptive brown recluse spider bite. *J Toxicol Clin Toxicol*. 1994;32(4):451-6. doi:10.3109/15563659409011048. |
| Nance WE. Hemolytic anemia of necrotic arachnidism. *Am J Med. 1961;31:801-7. d*oi:10.1016/0002-9343(61)90164-4. |
| Neverman EM, Vietti D, Gruner B. Fever, jaundice, and confusion. *Clin Pediatr (Phila). 2017;*56(1):90-92. *d*oi:10.1177/0009922816678978. |
| Nguyen N, Pandey M. Loxoscelism: Cutaneous and Hematologic Manifestations. *Adv Hematol*. 2019;2019:4091278. doi:10.1155/2019/4091278. |
| Nicholson JF, Nicholson BH. Hemolytic anemia from brown spider bite: Necrotic arachnidism. *J Okla State Med Assoc*. 1962;55:234-236. |
| Norris K, Misra S. Brown recluse spider bite on the breast. *JAAPA*. 2014;27(5):32-34. doi:10.1097/01.JAA.0000443967.31234.87. |
| Novak R, Mehesh Kumar AP, et al. Severe systemic toxicity from a spider bite in a 6-year-old boy. *J Tenn Med Assoc*. 1979;72:110-111. |
| Nunnelee JD. Brown recluse spider bites: a case report. *J Perianesth Nurs. 2006*;21(1):12-5. doi:10.1016/j.jopan.2005.08.006. |
| Perry WJ. The brown recluse spider in Alabama: a report of five cases. *J Med Assoc State Ala*. 1975;44(10):551-555. |
| Ramirez FE, Nedley N. Neutralizing the toxin of the brown recluse (*Loxosceles reclusa*) spider with activated charcoal poultices. *Drug Metab Rev*. 2016;48. |
| Ratakonda B, Trabue C. A rare case of Coombs-positive hemolytic anemia due to brown recluse spider bite. *Am J Respir Crit Care Med*. 2020;201. |
| Raza S, Shortridge JR, Kodali MK, et al. Severe haemolytic anaemia with erythrophagocytosis following the bite of a brown recluse spider. *Br J Haematol.* 2014;167(1):1. doi:10.1111/bjh.13019. |
| Rees RS, Altenbern DP, Lynch JB, et al. Brown recluse spider bites. A comparison of early surgical excision versus dapsone and delayed surgical excision. *Ann Surg*. 1985;202(5):659-663. doi:10.1097/00000658-198511000-00020. |
| Riley HD, McLean WR, Start H, et al. Brown spider bite with severe hemolytic phenomena. *J Okla State Med Assoc*. 1964;57:218-223. |
| Robb CW, Hayes BB, Boyd AS. Generalized vasculitic exanthem following *Loxosceles reclusa* envenomation. *J Cutan Pathol*. 2007;34(6):513-514. doi: 10.1111/j.1600-0560.2006.00657.x. |
| Roberts K, DeLeon S. Falsely reassured: later onset hemolytic anemia in loxescelism. *Am J Med Sci. 2024;367.* |
| *R*ose NJ. Report of fatality: spider bite (Loxosceles). *IMJ Ill Med J*. 1970;137(4):339. |
| Rubin RN. A painful isolated skin lesion on a man’s shoulder. *Consultant*. 2018;58(6):e185. |
| Ruelle AL, Sowell ME, Derk FF, et al. Multiple brown recluse spider envenomation. *J Am Podiatr Med Assoc*. 1996;86(4):174-176. doi:10.7547/87507315-86-4-174. |
| Said A, Hmiel P, Goldsmith M, et al. Successful use of plasma exchange for profound hemolysis in a child with loxoscelism. *Pediatrics*. 2014;134(5):e1464-e1467. doi:10.1542/peds.2013-3338. |
| Sams HH, Hearth SB, Long LL, et al. Nineteen documented cases of *Loxosceles reclusa* envenomation. *J Am Acad Dermatol*. 2001;44(4):603-608. doi:10.1067/mjd.2001.112380. |
| Sauer GC. Transverse myelitis and paralysis from a brown recluse spider bite. *Mo Med*. 1975;72(10):603-604. |
| Schilli KD, Rader RK, Payne KS, et al. Obtundation and myocardial infarction in a case of systemic loxoscelism. *Mo Med*. 2014;111(2):143-147. |
| Schmid KM, Treaster MR, Barrios C, et al. Heightened immune response to presumed *Loxosceles reclusa* envenomation. Wilderness Environ Med. 2019;30(4):450-453. doi:10.1016/j.wem.2019.06.014. |
| Sharma S., MacKay K, Vedantam K, et al. A rare case of severe loxoscelism causing acute hemolytic anemia with superimposed COVID-19 infection. *J Gen Intern Med*. 2023;38(Suppl 2). |
| Simmons R, Curtin JW. Brown recluse spider bite - increasing incidents in northern Illinois. *Ill Med J*. 1974;145(1):43-46. |
| Simpson M, Williams S. Acute generalized exanthematous pustulosis associated with loxosceles spider bite. *Clin Toxicol*. 2020;58(11):1221. |
| Sims RA, Fish-Trotter HL, Clark DE, et al. Toxin-mediated myocarditis from a brown recluse spider bite. *JACC Case Rep*. 2021;4(1):49-53. doi:10.1016/j.jaccas.2021.10.003. |
| Sood SB, Banner W, Barton RP. Extracorporeal cardiopulmonary resuscitation after brown recluse envenomation. *Clin Toxicol* (Phila). 2017;55(5):368-389. doi:10.1080/15563650.2017.1287913. |
| Stoecker WV, Green JA, Gomez HF. Diagnosis of loxoscelism in a child confirmed with an enzyme-linked immunosorbent assay and noninvasive tissue sampling. *J Am Acad Dermatol*. 2006;55(5):888-890. doi:10.1016/j.jaad.2006.04.065. |
| Stoecker WV, Wasserman GS, Calcara DA, et al. Systemic loxoscelism confirmation by bite-site skin surface: ELISA. *Mo Med*. 2009;106(6):425-427, 431. |
| Svendsen FJ. Treatment of clinically diagnosed brown recluse spider bites with hyperbaric oxygen: a clinical observation. *J Ark Med Soc*. 1986;83(5):199-204. |
| Talib U, Abdelfattah AH, Talib M, et al. Brown Recluse Spider Bite Resulting in Coombs Negative Hemolytic Anemia in a Young Male Requiring Blood *Transfusion*. *Cureus*. 2022;14(7):e26574. doi:10.7759/*cureus*.26574. |
| Taylor EH, Denny WF. Hemolysis, renal failure and death, presumed secondary to bite of brown recluse spider. *South Med J*. 1966;59(10):1209-1211. doi:10.1097/00007611-196610000-00021. |
| Taylor MA, Olive AT. Brown recluse spider bite. Report of a case. *N C Med J*. 1972;33(5):421-424. |
| Truong TV, Gruenberg B, Ciener DA, Butchee R. Hives and fever in a 13-year-old boy. *Pediatr Rev*. 2022;43(1):49-53. doi:10.1542/pir.2020-003848. |
| Vinayek N, Kloecker GH, Riley BC. Acquired hemophilia after a spider bite. *Blood*. 2012;120(21):4637. doi:10.1182/blood.V120.21.4637.4637. |
| Vorse H, Seccareccio P, Woodruff K, et al. Disseminated intravascular coagulopathy following fatal brown spider bite (necrotic arachnidism). *J Pediatr*. 1972;80(6):1035-1037. doi:10.1016/s0022-3476(72)80023-4. |
| Wagner SJ, Ricke BJ, Rianprakaisang TN, et al. Idiopathic intracranial hypertension in confirmed case of systemic loxoscelism. *J Med Toxicol*. 2020;16(2):160-161. |
| Walker JS, Hogan DE. Clinical Pearls Bite to the Left Leg. *Acad Emerg Med*. 1995;2:223. doi:10.1111/j.1553-2712.1995.tb03204.x. |
| Way S, Lachar G, Givens M. A child with fever, malaise, and a skin wound. *Pediatr Emerg Care*. 2005;21(9):620-623. |
| Wesley RE, Ballinger WH, Close LW, et al. Dapsone in the treatment of presumed brown recluse spider bite of the eyelid. *Ophthalmic Surg*. 1985;16(2):116-117, 120. |
| Williams ST, Khare VK, Johnston GA, et a. Severe intravascular hemolysis associated with brown recluse spider envenomation. A report of two cases and review of the literature. *Am J Clin Pathol.* 1995;104(4):463-467. doi:10.1093/ajcp/104.4.463. |
| Wilson JR, Hagood CO Jr, Prather ID. Brown recluse spider bites: a complex problem wound. A brief review and case study. *Ostomy Wound Manage*. 2005 Mar;51(3):59-66. |
| Wright SW, Wrenn KD, Murray L, et al. Clinical presentation and outcome of brown recluse spider bite. *Ann Emerg Med*. 1997;30(1):28-32. doi:10.1016/s0196-0644(97)70106-9. |
| Yi X, AuBuchon J, Zeltwanger S, et al. Necrotic arachnidism and intractable pain from recluse spider bites treated with lumbar sympathetic block: a case report and review of literature. *Clin J Pain*. 2011;27(5):457-460. doi:10.1097/AJP.0b013e31820b6424 |
